# Short-Term Change in Occlusal Function after Using Mandibular Advancement Appliance for Snoring: A Pilot Study

**DOI:** 10.1155/2012/652154

**Published:** 2012-04-08

**Authors:** Hiroshi Ueda, Genki Watanabe, Atsushi Horihata, Myongsun Koh, Kazuo Tanne

**Affiliations:** Department of Orthodontics and Craniofacial Developmental Biology, Graduate School of Biomedical Sciences, Hiroshima University, 1-2-3 Kasumi, Minami-Ku, Hiroshima 734-8553, Japan

## Abstract

The main aim was to evaluate the influence on occlusal contact area (OCA), maximum bite force (MBF), center of occlusal load (COL), and tooth pain after the nocturnal use of different mandibular advance appliances (MAAs) for snoring. Subjects were consisted of ten adult volunteers with mild snoring in Hiroshima University Hospital. Recordings of occlusal function were performed six times for two hours, that is, immediately and 5, 15, 30, 60, and 120 minutes after the nocturnal use of MAA. The subjects continuously scored their pain intensity on a 10 cm visual analogue scale (VAS) when MBF was measured. Comparing two MAAs, OCA and MBF were significantly larger in two-piece MAA than in one-piece MAA five minutes after removing the appliance. Significant difference in COL and VAS score compared to baseline disappeared more quickly with two-piece MAA than with one-piece MAA. In conclusion, it is shown that two-piece MAA could be superior to the one-piece one in terms of the degree side effect on occlusal function.

## 1. Introduction

Obstructive sleep apnea (OSA) is characterized by repetitive episodes of upper airway obstruction that occur during sleep, usually associated with a reduction in blood oxygen saturation [1]. Excessive daytime sleepiness caused by nocturnal sleep fragmentation interferes with daytime activities, being a common complaint of patients with OSA. OSA is also associated with increased morbidity and mortality from cardiovascular, metabolic, and cognitive alterations in adults [2–7].

Mandibular advancement appliances (MAAs), aiming to enlarge the upper airway by repositioning the mandible forward, are known to be useful as a lifelong treatment tool for primary snoring and mild-to-moderate OSA [8–11]. MAAs are sometimes more preferable for moderate-to-severe OSA when the nasal continuous positive airway pressure (nCPAP) therapy is not indicated, although MAA therapy was less effective than nCPAP [12, 13].

Recently, several studies have focused on the side effects of MAAs related to temporomandibular joint (TMJ) discomfort and the masticatory muscles stiffness with difficulty in chewing immediately after the nocturnal use of MAAs [14–16]. George [17] reported that most OSA patients, after the use of MAA for a whole night, experience that the bite does not feel right upon awakening, but that it normalizes after breakfast. Ringqvist et al. [18] also demonstrated that some patients had problems in biting in the regular habitual intercuspal position during the first hour or so after using MAA for a whole night. The adverse event might be small and transient, but it happens repeatedly every morning.

To date, numerous MAAs are available and distributed in the market for the treatment of snoring and OSA [19]. MAAs can be classified roughly into two different types. One is one-piece type and the other is two-piece one. Especially, the latter is expected to improve jaw and muscle discomfort during MAA therapy. However, side effects of two-piece type MAA on masticatory system have not been investigated objectively and comparatively.

 In the present study, a controlled randomized study was designed to test a hypothesis that the influence on occlusal function and tooth pain would be less in two-piece MAA than in one-piece ones. The primary aim was to compare the difference in mandibular movement of two-type MAAs when one-piece and two-piece MAAs are similarly used. Secondary aim was to evaluate the influence on occlusal contact, bite force, and tooth pain after the nocturnal use.

## 2. Materials and Methods

### 2.1. Subjects

Subjects were consisted of five males and five females (mean age: 26.2 ± 5.8 years) selected from the volunteers who have mild snoring in Department of Orthodontics, Hiroshima University Hospital. The inclusion criteria for the subjects were as follows: (1) normal horizontal and vertical skeletal relationships, (2) no malocclusions and periodontal disease, (3) no complaints of TMJ disorders, and (4) no history of snoring treatment.

Informed consent was obtained from each subject prior to the experiment. The Ethics Committee of Hiroshima University approved the secondary use of data in this study.

### 2.2. Measurement of Mandibular Movement with Different MAAs

**Figure 1:**
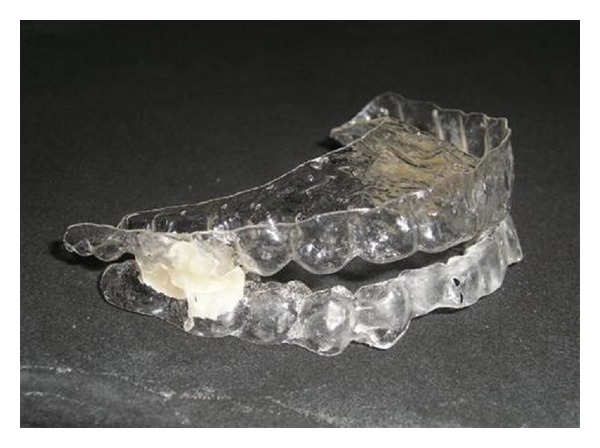
One-piece MAA.

**Figure 2:**
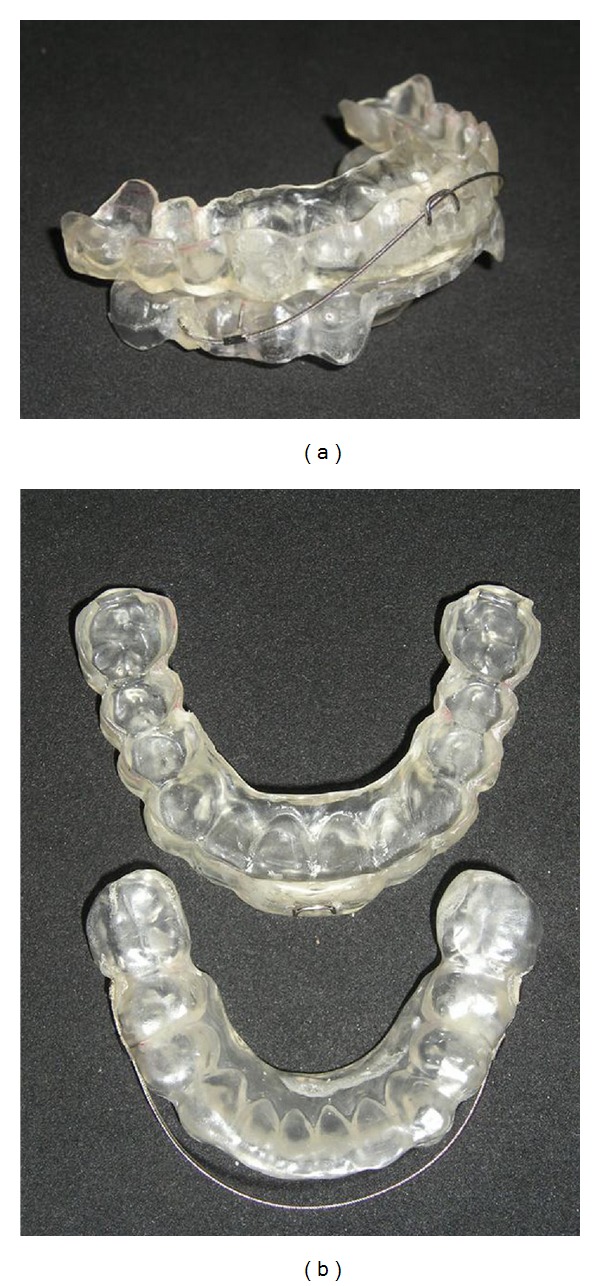
(a) Two-piece MAA (lateral view), (b) upper and lower plates.

Both one-piece (Figure 1) and two-piece (Figures 2(a) and 2(b)) MAAs were constructed of 0.75 mm thick acrylic resin that provides full occlusal coverage of teeth. Two-piece MAA is made of resin and an orthodontic wire, 0175′′ multistranded round wire, attached on the buccal sides of the lower splint. The amount of initial mandibular advancement achieved by both MAAs was defined as two-thirds of maximum mandibular forward repositioning with a 3-4 mm vertical opening in the anterior teeth. The mean advanced mandibular position was very similar, 6.2 ± 0.9 mm and 6.4 ± 1.0 mm with one-piece and two-piece MAAs, respectively. The appliance was adjusted to maximize comfort by relieving all uncomfortable pressure points on the teeth and gums.

**Figure 3:**
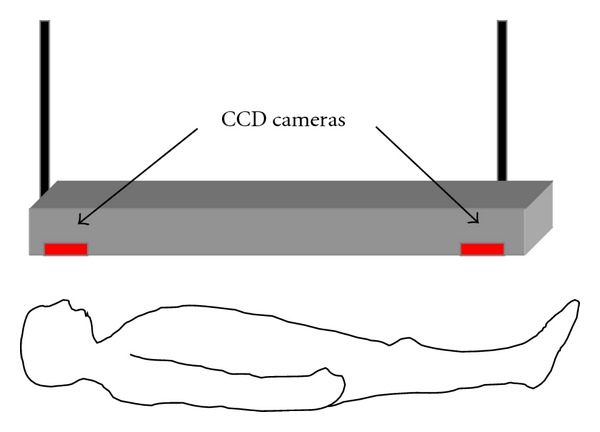
Schematic illustration to show the recording jaw movement by means of Gnathohexagraph.

Prior to the initiation of one-night clinical study, the difference in the maximum mandibular movement between using both MAAs was investigated horizontally in the anteroposterior (P-A) and right-left (R-L) directions by means of an optoelectric jaw-tracking system with six degrees of freedom. This system consists of a head frame, face bow, light-emitting diodes (LEDs), two CCD cameras, and a personal computer (Gnathohexagraph system II, Onosokki Co., Yokohama, Japan) [20, 21]. Each subject was in supine position on the floor with a head frame and a face bow, each with 3 LEDs. These devices were attached firmly to the head and the dental clutch, which was bonded to the labial surface of the lower splints. Two CCD cameras were placed approximately 1.2 meters away from subjects, as illustrated in Figure 3.

Each subject was requested to perform maximum voluntary movements with MAA three times, all the way to reposition the jaw forward and backward, and side to side. We calculated the maximum displacement of the mandible from the path of LEDs recorded by CCD cameras.

### 2.3. Clinical Recording and Analytical Procedure

**Figure 4:**
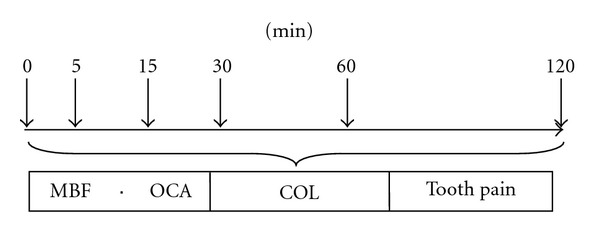
Schematic illustration to show the time course.

The subjects were randomized to use either one-piece or two-piece MAA for six or more hours for a whole night and crossed over to the other MAA after one-week washout period. Recordings of occlusal function were performed six times for two hours, that is, immediately and 5, 15, 30, 60, and 120 minutes after the nocturnal use of MAA. The sequence of recordings and evaluations is illustrated in Figure 4.

### 2.4. Evaluation of Occlusal Function

**Figure 5:**
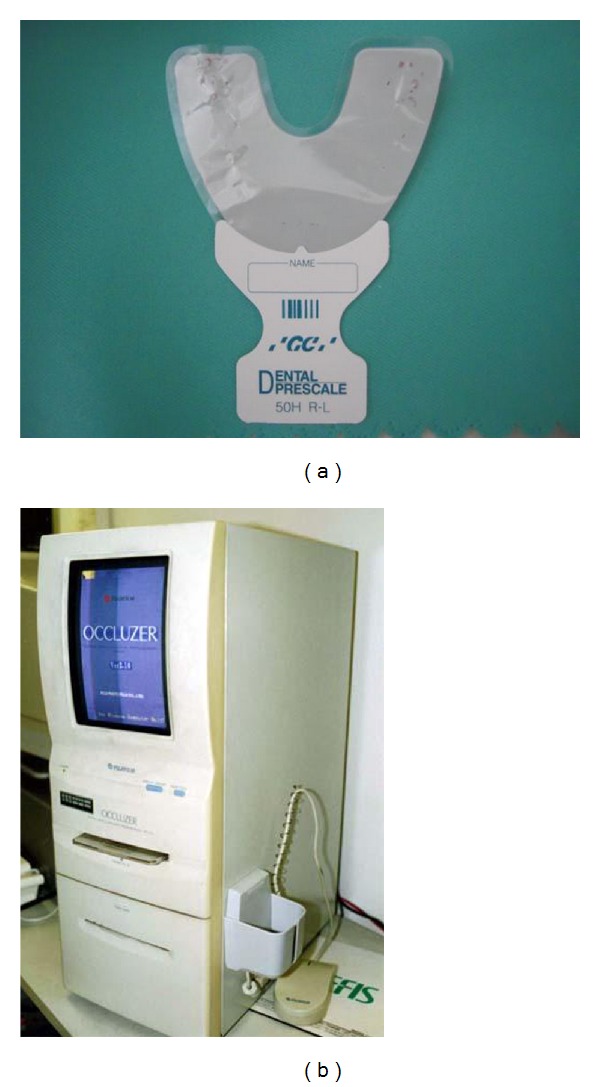
(a) A pressure-sensitive sheet (dental prescale), (b) an occlusal diagnostic system (dental prescale occluzer).

**Figure 6:**
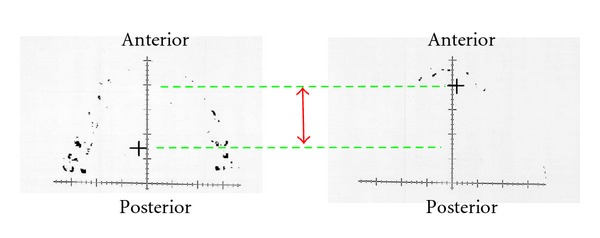
A cross represents the COL in the upper dentition during maximum clenching.

An occlusal diagnostic system, Dental Prescale Occluzer, (Fuji Film Co., Tokyo, Japan) was used to evaluate occlusal contact area (OCA), maximum bite force (MBF), and center of occlusal load (COL) (Figures 5(a) and 5(b)) [22–26]. Dental Prescale is a 98 *μ*m thick horseshoe-shaped sheet wrapped with polyfilm. The microcapsules in the sheet break and release a color-forming material at various occlusal pressures. Each subject was instructed to bite a pressure-sensitive sheet in the habitual intercuspal position with maximum clenching for 3 seconds by one of the investigators (GW). Then, an image scanner (FPD-703, Fuji Film Co.) was used to determine OCA, MBF, and COL. Both OCA and MBF were quantified with an occlusal force diagram according to the degree of coloring. In order to evaluate changes in COL, the anteroposterior distance from the baseline point is measured (Figure 6).

### 2.5. Visual Analogue Scale for Tooth Pain

**Figure 7:**
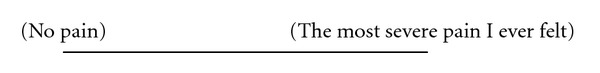
Visual analogue scale for tooth pain.

The volunteers continuously scored their pain intensity on a 10 cm visual analogue scale (VAS) when MBF was measured. The upper extreme was marked “most pain imaginable,” and the lower extreme was marked “no pain” (Figure 7).

VAS scores were calculated as tooth pain in accordance with previously described methods [27, 28].

### 2.6. Statistics

The arithmetic mean and standard deviation (SD) were calculated for each variable. No significant differences were found between sexes. Recordings from men and women were therefore pooled and analyzed together.

Statistical significance of the differences in the measured values between the MAAs was performed with Mann-Whitney *U* test. Among six performances, the data were statistically analyzed using analysis of variances (ANOVA) and the subsequent pairwise comparisons.

*P* values less than 0.05 were considered significant.

## 3. Results

### 3.1. Maximum Mandibular Movement with MAA

Table 1 shows the comparison of maximum mandibular movement when two-type MAAs were used. Two-piece MAA showed a greatly significant R-L movement, approximately 11 mm in the transverse direction, during voluntary lateral jaw movement.

With respect to the P-A direction, significant differences were found between two MAAs during maximum jaw opening and lateral movement. However, mean displacement of the mandible using two-piece MAA was below 2 mm.

### 3.2. Evaluation of Occlusal Function and Tooth Pain

**Figure 8:**
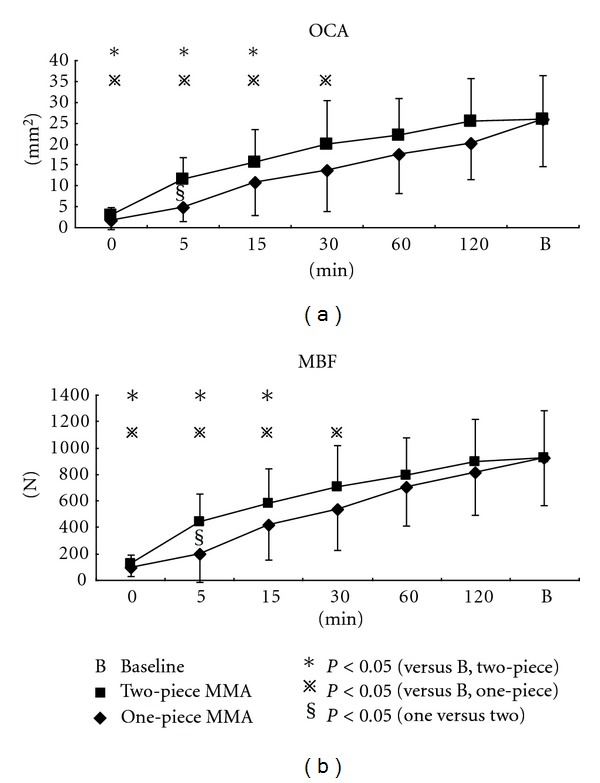
(a) Changes in occlusal contact area after nocturnal use of different MAAs, (b) changes in maximum bite force after nocturnal use of different MAAs.

Changes in OCA and MBF after nocturnal use of two MAAs are shown in Figures 8(a) and 8(b), respectively. OCA is increased gradually from approximately 2.5 to 16 mm^2^ during 120-minute measurement. MBF exhibited similar changes to OCA and showed an increase gradually from approximately 100 to 800 N through the experiment. Thirty and fifteen minutes after initiating the measurements, OCA and MBF were found significantly smaller than those of the baseline in one-piece and two-piece MAAs, respectively. After 30 minutes, significances in OCA and MBF disappeared, although both two parameters still showed almost 80% of baseline after 120 minutes.

Comparing two MAAs, OCA and MBF were significantly larger in two-piece MAA than in one-piece five minutes after removing the appliance.

**Figure 9:**
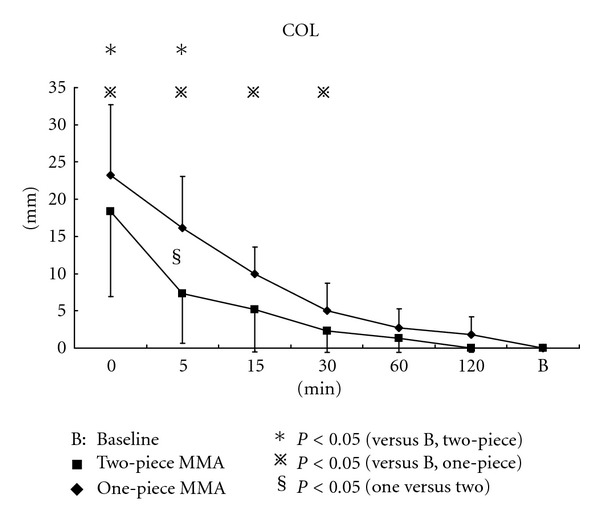
Changes in center of occlusal load after nocturnal use of different MAAs.

Mean COL after using MAA for a whole night is located approximately 20 mm anterior (around the canines or first premolars) to the baseline COL (Figure 9). Then, it is getting closer to the baseline point (around the first molars) shifting backward through the morning.

Significant difference in COL compared to baseline disappeared more quickly with two-piece MAA than with one-piece MAA, after 5 and 30 minutes, respectively.

Comparing two MAAs, COL was significantly larger in one-piece MAA than in two-piece five minutes after removing the appliance. Although COL returned to the baseline 120 minutes after the use of two-piece MAA, COL with one-piece MAA was still 3 mm backward from the baseline.

**Figure 10:**
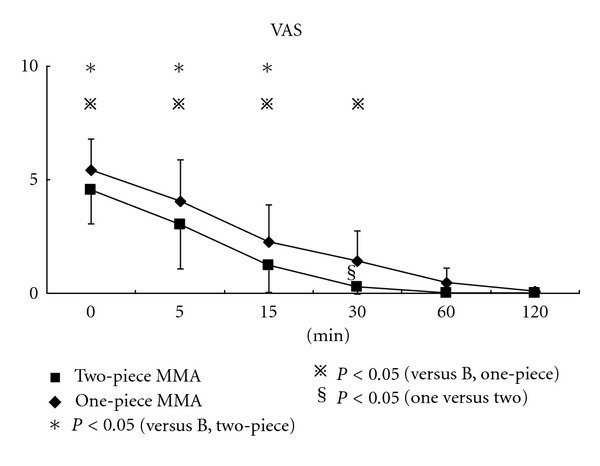
Changes in tooth pain after nocturnal use of different MAAs.

Figure 10 shows changes in VAS for tooth pain with two MAAs. Mean VAS score immediately after removing MAA was between 5 and 6. Significant difference in VAS score compared to baseline disappeared more quickly with two-piece MAA than with one-piece MAA, after 15 and 30 minutes, respectively.

Comparing two MAAs, VAS score was higher in one-piece MAA than in two-piece MAA until 30 minutes, and significance was found 30 minutes after removing the appliance. After one hour, mean VAS decreased to below 1, and for two hours during the experiment, VAS score became the same as the baseline, that means 0 (no pain).

## 4. Discussion

This study is the first prospective randomized crossover study to compare influence of two-type MAAs masticatory system. Two-piece MAA showed a significantly greater jaw movement in the transverse direction. However, with respect to the P-A direction, mean displacement of the mandible was below 2 mm during any jaw movement in the laboratory-based results. Furthermore, two-piece MAA which allows mandibular movement in various directions showed significantly less influence on occlusal function and tooth pain than one-piece MAA.

Currently, more than 50 different devices are distributed in the market for dentists in USA and Canada [29], where one-piece MAA has been used most frequently because it is cheaper and easier to fabricate than two-piece, adjustable appliances. However, two-piece type has become popular because of the merit to decrease orofacial discomfort.

In the present study, all subjects with either MAA got considerable improvement of snoring based on the information from their bedpartners. Thus, the efficacy of two different MAAs was clinically similar. Different MAAs have been designed and evaluated in previous studies. Ferguson et al. [30] reported two prospective randomized crossover studies to compare the efficacy, side effects, and preference of both rigid and nonrigid MAAs relative to nCPAP. Both MAAs were effective in reducing symptoms, and the long-term preference was overwhelmingly in favor of MAA therapy, superior to nCPAP. Treatment success rate was 48% and 61%, and compliance failure rate was 24% and 4% with nonadjustable and adjustable MAAs, respectively in their different studies. In addition, another study [16], directly compared a one-piece appliance with a two-piece one. Fritsch et al. showed no significant difference, but one-piece, monoblock MAA was comparatively favored by patients because it produces negligible side effects.

As far as we know, no clinical randomized and controlled study has confirmed objectively the superiority of two-piece MAA over one-piece one in terms of the influences on masticatory system. In this study, mean OCA and MBF were significantly small, approximately 20% of baseline immediately after the nocturnal use of both the types of MAAs, then getting increased gradually during two hours. Significant differences from the baseline were found until half an hour passed.

Otsuka et al. [31] found that mean OCA and MBF of OSA patients during MAA therapy were significantly smaller in the morning than at night. They also demonstrated a decrease in the occlusal function by 40% when morning and night data were compared. Ueda et al. [32] also reported the similar findings in consecutive 3-day data in OSA patients.

Both OCA and MBF were significantly decreased in the morning. The finding may be explained in part by the side effects of MAA in use such as joint edema, stiffness of masticatory muscles, or altered occlusal function [30, 33]. In addition, the most likely explanation for the reduction in occlusal function immediately after the nocturnal use of MAA is that the mandible is repositioned anteriorly by MAA and does not return to the original normal position, which means the condyle cannot attain its normal position during maximum clenching. One important result of this study was that COL with two-piece MAA returned the baseline point significantly quickly. COL data in this study support our speculation because significant forward COL means less occlusal contact in the molar region and close contact in the anterior region, due to MAA-induced forward position of the mandible.

 The recovery of OCA and MBF was observed up to 120 minutes after removal of MAA, although occlusal function could not reach the baseline level yet for all the subjects. The fact that a few subjects presented reduced occlusal function by 50% below the baseline with one-piece MAA even two hours after the removal should not be neglected.

When the mandible is repositioned forward rigidly even several hours per night, a major concern has been that it may generate a special strain upon the orofacial structures, causing adverse effects on the TMJ and masticatory muscles. Masticatory system including the mouth, pharynx, and TMJ is involved cooperatively in many vital physiological activities, such as breathing, swallowing saliva, and yawning during sleep. Miyamoto et al. [34] investigated mandibular posture during sleep in seven healthy adults and OSA patients. Their results showed that vertical mandibular posture is more downward during sleep in OSA patients than in controls, and mandibular opening progressively increases from 0 to more than 10 mm during apnoeic episodes. In our previous study, Shikata et al. [35] found that the amount and duration of vertical mandibular displacement were significantly increased (approximate 10 mm) by experimentally induced nasal respiratory obstruction in awake healthy subjects. From these studies, the nature of mandibular displacement with breathing disorder can be often observed at around 10 mm mouth opening; therefore, we believe that it is more favorable not to fix the mandible rigidly in terms of the jaw reflex and masticatory stiffness even during sleep.

Another explanation for the superiority of two-piece MAA may be that the compliance of using MAA relate to the balance between the benefits and side effects [36]. de Almeida et al. [37] demonstrated 35.9% of the 251 OSA patients stopped MAA treatment after a mean of 5.7 years and 72% of those did so during the first year of treatment. In their speculation, major reasons that patients discontinued MAA treatment were major side effects and poor treatment effects on snoring and OSA symptoms. With these considerations, it is of a great importance to diminish side effects of MAA and to enhance the treatment effects for snoring and OSA to be satisfied by patients.

VAS is used extensively as a subjective estimation for pain. That is quite simple and easy for subjects. As a result of VAS examination, the recover of tooth pain was a little different from occlusal function mentioned above. After two hours, tooth pain during biting the pressure-sensitive sheet disappeared totally on VAS. This may be due to that the threshold of human sensation has some range. In addition, tooth pain might be easy to be influenced easily by individual's feeling under psychological situation.

The present study has several potential limitations. Subjects in this one-night study were young volunteers with mild snoring, not diagnosed OSA patients. Further investigation in this area is required, especially for typical middle-aged OSA patients who have disadvantage of periodontium undergoing MAA treatment with one-piece or two-piece type. The influence of long-term use of MAA on masticatory system is to be examined in more detail.

Based on the results, side effects on occlusal function might be transient, but unstable mandibular position for a long time in every morning could predispose snoring and OSA patients to the permanent occlusal change. In conclusions, two-piece MAA that can allow jaw movement in various directions might avoid excessive stiffness of masticatory muscles and distribute the harmful force widely on the dentition. Thus, it is shown that two-piece MAA could be superior to the one-piece one in terms of the degree of the side effects on occlusal function.

## Figures and Tables

**Table 1:** The distance of mandibular movement with different MAAs during maximum voluntary effort.

		One-piece MAA	Two-piece MAA	
Right-left direction	Shift to the right	0.64 ± 0.41	11.1 ± 2.49	**
	Shift to the left	0.79 ± 0.67	11.4 ± 1.83	**
Anteroposterior direction	Maximum jaw opening	0.51 ± 0.38	1.71 ± 0.79	NS
	Lateral jaw movement	0.32 ± 0.08	1.00 ± 0.66	NS

Unit: mm, **P < 0.01, NS: not significance.
